# Procedures and timetable for proposals to amend *Chapter F* of the *International Code of Nomenclature for algae, fungi, and plants*

**DOI:** 10.1186/s43008-020-00044-w

**Published:** 2020-09-24

**Authors:** Tom W. May

**Affiliations:** 1 Royal Botanic Gardens Victoria, Birdwood Avenue, Melbourne, Victoria 3004 Australia; 2 Fungal Nomenclature Bureau, XII International Mycological Congress, Amsterdam, The Netherlands

**Keywords:** Fungal Nomenclature Bureau, Fungal Nomenclature Session, Governance, International Mycological Association, International Mycological Congress, *San Juan Chapter F*

## Abstract

Procedures for preparing and submitting proposals to amend or enhance *Chapter F* of the *International Code of Nomenclature for algae, fungi, and plants* are provided. Such proposals will be considered by the Fungal Nomenclature Session of the XII International Mycological Congress to he held in Amsterdam in 2022. A timetable is laid out for the submission of proposals, due by 31 December 2021, their publication in *IMA Fungus*, the appearance of the ‘Synopsis of proposals” and the conduct of the pre-Congress guiding vote.

## INTRODUCTION

In the *International Code of Nomenclature for algae, fungi, and plants* (*Code*), the current edition of which is the *Shenzhen Code* (Turland et al. 2018), *Chapter F* brings together provisions specific to fungi. Material in *Chapter F* falls under the governance of the International Mycological Congress (IMC) (See Table 1). The *Code* specifies (Division (Div.) III, Provision (Prov.) 1.2, 8.1) that *Chapter F* may be modified only by action of a plenary session of an IMC on a resolution moved by the Fungal Nomenclature Session (FNS) of that Congress. The current *Chapter F* is the *San Juan Chapter F*, published on 27 December 2019 (May et al. 2019). It incorporates changes to the previous version of *Chapter F* that was part of the *Shenzhen Code* (Turland et al. 2018) in which a separate *Chapter F* was first introduced (Hawksworth et al. 2017). The changes were adopted on 21 July 2018 at IMC11 in San Juan, Puerto Rico. The next International Mycological Congress (IMC12) is scheduled to be held in Amsterdam, The Netherlands, in August 2022, with the Fungal Nomenclature Session occurring during the Congress.

**Table 1:** Key organizations and events related to the governance of fungal nomenclature

• *International Code of Nomenclature for algae, fungi, and plants* (*Code*)	
• *Chapter F —* the section of the *Code* containing provisions pertaining solely to fungi	
• Fungal Nomenclature Bureau (FNB) *—* runs the FNS at an IMC	
• Fungal Nomenclature Session (FNS) *—* considers proposals to amend or enhance *Chapter F*	
• Guiding vote *—* a non-binding indication of opinions on the proposals; held prior to the FNS	
• International Mycological Association (IMA)	
• International Mycological Congress (IMC) *—* organized by the IMA; decisions of the FNS are ratified during its plenary session	
• Nomenclature Committee for Fungi (NCF) *—* considers proposals to conserve or reject names of fungi; advises on but does not decide on proposals to amend *Chapter F*	

Procedures of the FNS are laid out in Div. III Prov. 1, 2, 4, 5 and 8 of the *Code*, and closely follow procedures of the Nomenclature Section of an International Botanical Congress (IBC) with the exception that there are no institutional votes at an IMC FNS. The Fungal Nomenclature Bureau (FNB) of an International Mycological Congress is responsible for running the FNS and the pre-Congress guiding vote. The FNB consists of various members including the Secretary, who is equivalent of the Rapporteur-général in the Bureau of Nomenclature of an IBC (May and Redhead 2018). Procedures for appointment of the officers of the FNB are set out in Div. III Prov. 4 and 8. The Secretary for each IMC is elected at the previous IMC. At IMC11 in San Juan, Tom W. May was elected Secretary of the FNB for IMC12 (May et al. 2018).

## PROCEDURE FOR AMENDING *CHAPTER F* OF THE *CODE*

The procedure for proposing changes to *Chapter F* of the *Code* is as follows:

- After each new version of *Chapter F* appears, proposals to amend it are published in *IMA Fungus*, where they are numbered serially. Such proposals may suggest rewording of existing Articles or Recommendations of *Chapter F* and/or introduce new material dealing solely with names of organisms treated as fungi (including fossil fungi).
- Approximately 4 months prior to the next International Mycological Congress, a “Synopsis of proposals” assembles all published proposals to amend *Chapter F*, organized by Article and Recommendation, and republishes them in *IMA Fungus*. The synopsis does not include the justification accompanying the original publication but is a commentary from the Secretary and Deputy Secretary of the FNB from a technical standpoint — especially in relation to clarity of wording, ramifications for other articles and unexpected consequences. The synopsis also includes the opinions of relevant bodies such as the Nomenclature Committee for Fungi (NCF).
- A pre-Congress guiding vote is held online, commencing soon after the synopsis is published. The guiding vote is a non-binding indication of the opinions of those individuals entitled to vote, as set out in Div. III Prov. 8.3 — essentially members of the International Mycological Association (IMA), authors of proposals and members of the NCF (May and Miller 2018). Results of the guiding vote are made available on the IMA website prior to the FNS of the Congress.
- The Fungal Nomenclature Session, meeting during the Congress, considers the published proposals, including any amendments to them. For each proposal, there is a formal vote among those individuals present on the day.
- Decisions of the FNS are ratified by vote of the plenary session of the Congress. At this point, the amended provisions take effect.
- The new edition of *Chapter F* is published. The online version of the *Code* is amended with a note pointing to the new edition of *Chapter F*.

## TIMETABLE AND RULES FOR PROPOSALS

The following timetable and regulations shall apply to proposals submitted to *IMA Fungus* for consideration by the Fungal Nomenclature Session of the XII International Mycological Congress in Amsterdam (See Table 2):

- *IMA Fungus* will open for proposals beginning now.
- The closing date for submission of proposals is 31 December 2021.
- Proposals may be externally reviewed.
- Late submissions received by the end of January 2022 may be accepted if no reviewing or major editing is necessary.
- Proposals are to be submitted by e-mail to the Secretary of the FNB (tom.may@rbg.vic.gov.au).
- After editing, proposals will be made available on the website of the IMA (http://www.ima-mycology.org/nomenclature/formal-proposals) and published in *IMA Fungus* in the Nomenclature category.
- Depending on the number of proposals, their length, and the time at which they are submitted, individual proposals and their accompanying justifications may be published separately or bundled together for publication as occurred prior to the last IMC (Hawksworth 2018).
- Proposals to amend *Chapter F* at the Amsterdam IMC in 2022 must be based on the text of the *San Juan Chapter F* (May et al. 2019).
- Manuscripts must conform to the general guidelines of *IMA Fungus* (see “Submission Guidelines” at https://imafungus.biomedcentral.com/submission-guidelines and in particular https://imafungus.biomedcentral.com/submission-guidelines/preparing-your-manuscript/nomenclature).
- There is no article-processing charge for items accepted for publication in the Nomenclature category of *IMA Fungus*.
- The rationale for a set of proposals must be presented concisely and may be condensed by the journal editors.
- If a proposal results from a more general context, authors must make that context the subject of a separate paper to be submitted to *IMA Fungus* (or some other journal), reviewed in the normal way, and considered for acceptance in competition with papers on other topics. Such manuscripts are to be submitted via the *IMA Fungus* online manuscript submission system (https://imafungus.biomedcentral.com/). The associated proposal, including a cross-reference to the relevant paper, will then be only as long as necessary and is to be submitted separately by e-mail to the Secretary of the FNB.
- In the case of a Special-purpose Committee, a report should be submitted to *IMA Fungus* that is a concise summary of the work of the Committee, providing context and justification for any associated proposals, which are to be prepared and submitted as above. Reports are to be submitted by e-mail to the Secretary of the FNB. There are two Special-purpose Committees commissioned to report to the XII International Mycological Congress: the Special-purpose Committee on DNA Sequences as Types for Fungi and the Special-purpose Committee on Names of Fungi with the Same Epithet. Membership of the Special-purpose Committees will be announced in the next Report of the Nomenclature Committee for Fungi, which will appear in *IMA Fungus*.
- The Editorial Committee can insert, delete, or change Examples and Glossary entries without a proposal (Div. III Prov. 7.11). Therefore, proposals to insert, delete, or change Examples in *Chapter F* or Glossary entries specific to provisions concerning fungi need not be published in *IMA Fungus* unless they are important for the understanding of a proposal to amend an Article, Note, or Recommendation. Instead, they may be submitted at any time directly to the Editorial Committee, which is represented prior to the Congress by the Secretary of the FNB (in relation to matters concerning fungi).

**Table 2:** Key dates for proposal to amend *Chapter F* of the *International Code of Nomenclature for algae, fungi, and plants*

Deadline for submission of proposals	31 December 2021
Publication of “Synopsis of proposals”	March 2022
Guiding vote	May 2022
Results of guiding vote	June 2022
Fungal Nomenclature Session (FNS) at IMC12	July 2022
Publication of revised “*Amsterdam Chapter F*”	Early 2023

## RECOMMENDATIONS FOR PROPOSALS

- Proposals should state explicitly what change is proposed, e.g.: “Insert a new Article after Art. F.10”; “Delete the last sentence of Art. F.8.1”; “Change Recommendation F.5A.1 to read as follows (new text in bold, deleted text in strikethrough)”. In other words, proposals should not consist simply of new text or explanation but should indicate exactly what text is to be *inserted*, *deleted*, or *changed* in *Chapter F*. A clearly expressed, concise proposal is more likely to be read sympathetically than a long complex argument or a series of repetitive proposals. Amendments to existing provisions of *Chapter F* can be precisely expressed by copying and pasting the provision and then showing new text in bold and deleted text in strikethrough.
- Proposals to amend the *Shenzhen Chapter F* submitted prior to the San Juan IMC and published in *IMA Fungus* serve as examples for layout and depth of coverage (Hawksworth 2018).
- Proposers are encouraged to provide, as part of the rationale for their proposal, some assessment of its impact to the stability of nomenclature. In general, proposals that contribute to nomenclatural stability are more likely to be successful.
- Proposals that address nomenclatural situations that occur only rarely are unlikely to succeed, especially if they add to the complexity of *Chapter F*. Such situations may be better addressed through conservation or rejection of a name, suppression of a work, a request for a binding decision, or a new Example in *Chapter F*.
- If a general principle applies to several Articles and Recommendations, the matter can often be covered by a single proposal, in which details are given at the most relevant point in *Chapter F*. If, for example, there should be a proposal to change the manner in which sanctioning is indicated, it would not be necessary to make a separate formal proposal for amending every single passage where sanctioned names appear. Similarly, if acceptance of a proposal would require renumbering or rewording of the remainder of an Article or Recommendation, such changes would automatically be made by the Editorial Committee, and it is not necessary to make separate proposals for them.
- Proposers are, however, encouraged to provide a list of all the Articles, Notes, Recommendations and Examples believed to be affected by a given proposal. This helps the Editorial Committee, whose main job is to ensure that any amendments adopted for the new *Chapter F* are fully integrated with the existing text and consistently implemented throughout.
- Examples should follow the format used in the *San Juan Chapter F*, i.e. with places of publication cited in full. Proposers should provide links to online versions of those publications and the protologues of any basionyms, replaced synonyms, or names causing illegitimacy. Online resources include the Biodiversity Heritage Library (https://www.biodiversitylibrary.org), Biblioteca digital del Real Jardín Botánico CSIC (https://bibdigital.rjb.csic.es), Gallica (https://gallica.bnf.fr/), and similar collections. For publications that are not yet online, a scanned copy of the relevant pages (including the title pages) is requested.

## PROPOSALS RELATED TO GOVERNANCE

Prior to the San Juan IMC FNS, some proposals were submitted relating to governance of fungal nomenclature, such as about the guiding vote and the “Fungal Editorial Committee”. At the FNS, these proposals were ruled as likely to be outside of the current mandate extended to the IMC because the new wording of the *Code* introduced at the Shenzhen IBC specifies that the mandate of the FNS covers “proposals relating to the content of Chapter F” (May et al. 2018, 2019). Because the rules about governance (including those relating to the FNS) are in Division III of the *Code* (rather than *Chapter F*) it seems that proposed amendments to governance of fungal nomenclature must be put before the Nomenclature Section of an IBC. According to Div. III Prov. 8.2, it is the “General Committee in consultation with the Nomenclature Committee for Fungi [that] is responsible for deciding which proposals relate solely to names of organisms treated as fungi”. This issue of whether or not mycologists can alter the rules about governance is the subject of ongoing discussions among the General Committee and the NCF. Whatever the outcome of these discussions, mycologists wishing to propose changes to governance, i.e. in relation to material in Div. III of the *Code*, are encouraged to submit proposals to the Secretary of the FNB following the procedures for proposals concerning *Chapter F* itself. If such proposals are deemed to be outside of the mandate of the FNS, an informal vote will nevertheless be taken, in order to inform subsequent debate should such proposals need to be introduced for consideration by the Nomenclature Section of the XX IBC, scheduled to be held in Rio de Janeiro, Brazil, in July 2023.

## PROPOSALS RELEVANT ALSO TO OTHER ORGANISMS

There may be proposals relevant to fungi that could well apply to all organisms that are covered by the *Code*, or to subsets of organisms wider than just fungi. If such proposals are submitted for consideration by the FNS of IMC12 they should be written so as to be limited to fungi. Proposals to amend the *Code* that are not specific to fungi should be submitted to the journal *Taxon* following the instructions provided by Turland and Wiersema (2019), by 31 March 2022, for consideration by the Nomenclature Section of the XX IBC.

## Data Availability

Not applicable.
